# A Community-Based Survey of Household Food Insecurity and Associated Sociodemographic Factors among 2–6 Years Old Children in the Southeast of Iran

**DOI:** 10.3390/nu13020574

**Published:** 2021-02-09

**Authors:** Mitra Sotoudeh, Sara Amaniyan, Mona Jonoush, Mojtaba Vaismoradi

**Affiliations:** 1 Iranshahr University of Medical Sciences, Iranshahr 7618815676, Iran; sotoude.m@irshums.ac.ir; 2 Student Research Center, Semnan University of Medical Sciences, Semnan 3514799422, Iran; s.amaniyan98@semums.ac.ir; 3 Department of Nutrition, Mashhad University of Medical Sciences, Mashhad 91778-99191, Iran; mona.jonoush@gmail.com; 4 Faculty of Nursing and Health Sciences, Nord University, 8049 Bodø, Norway

**Keywords:** food security, children, health, household, nutrition, underweight, stunting

## Abstract

Malnutrition remains a major public health issue in developing and transitional countries and food insecurity is a major indicator of the nutritional status in these societies. This research aimed to investigate the status of household food insecurity and sociodemographic factors affecting it among 2–6 years old children in an urban area in the southeast of Iran. A community-based survey was conducted from September to January 2018 on 421 children aged 2–6 years who were selected using stratified cluster random sampling. They lived in six different areas in an urban area in the southeast of Iran. Data was collected using the U.S. Department of Agriculture Food Security questionnaire and anthropometric measurements. Our study showed that the prevalence of food insecurity among children was 81.7% consisting of 2.6% with low food security, 9.2% with moderate food insecurity, and 69.9% with very low food security. The weight gain of those children who were in the low food security group, was 2.63 times lower than those children in the food security group. Moreover, the chance of weight gain in the low food security and in the moderate food insecurity groups was less as 1.91 and 1.41 times, respectively. Food insecurity in children aged 2–6 years is influenced by various sociodemographic factors including weight and height, mother’s education level, sanitation as access to water closet (WC). Policymakers should plan to improve the quality of life and health of the children through improving their food security.

## 1. Introduction

Food insecurity as a global challenge can lead to humanitarian catastrophes worldwide. The Food and Agriculture Organization of the United Nations (FAO) in 2009 stated that “for the first time since 1970, more than one billion people, about 100 million more than the last year, and around one-sixth of all of humanity, are esurient and malnourished worldwide” [1,2]. According to the joint annual report by the Food and Agriculture Organization of the United Nations (FAO), the International Fund for Agricultural Development, the United Nations International Children’s Emergency Fund (UNICEF), the World Food Program (WFP), and the World Health Organization (WHO), 690 million people in 2019 suffered from hunger [3].

Food insecurity is associated with starvation and destitution, and is considered a global universal public health problem with long-time negative consequences for children’s health. Moreover, it is a risk factor for child’s growth, because appropriate nutrition and adequate meal affect child’s well-being, physical, mental, and social development and his/her future health and welfare [4,5]. Those children who suffer from food insecurity are more likely to experience adulthood issues in terms of poor education outcomes and lower economic status [6]. It is believed that nutritious foods are required for proper growth and development. Sufficient calcium intake increases bone density, helps teenagers’ grow, and reduces the venture of hip fracture in later adulthood [7,8]. Therefore, the health, growth, and development of children are top priorities in all societies [9]. Concerns about food insecurity at the household level consist of an obvious association between food insecurity and poor health, insufficient food consumption, cognitive and academic decline, and psychosocial problems among children [10,11].

### Background in Iran

While some progress has been made to reduce the proportion of malnourished children over the past three decades across the globe, malnutrition remains a major public health issue in developing and transitional countries [12]. Some national strategies on food and nutrition security, including the national document for intersectoral food and nutrition security development, as well as the executive package of food and nutrition security, have been developed and implemented in Iran. They encompass three areas of sustainable food supply, food safety, and appropriate nutrition, but they have not achieved sufficient legitimacy and their effects on public health have remained unrecognized [13]. Probable reasons could be the lack of necessary infrastructure, insufficient cooperation of mid-level managers at medical sciences universities and health networks, and inattention to factors associated with food security in urban/rural areas [14,15].

Household food insecurity is known as a major factor influencing malnutrition. Therefore, food insecurity is a major indicator of the nutritional status in low-income societies [16]. A report showed that chronic malnutrition measured by stunting affected 32.5% of Iranian children [17]. According to the National Survey of Iranian Children, there is significant nutritional diversity in all parts of Iran, and the poorest indices have been reported in the southeast of Iran [18]. It has been estimated that 20–60% of households in Iran suffer from food insecurity and the inappropriate child food security status could be more common in poor provinces such as those located in the southeast of Iran [19]. However, there is a lack of high-quality and updated evidence regarding the condition of food insecurity based on community surveys in the southeast of Iran. Appropriate and comprehensive assessment of the state of food insecurity among households and the investigation of factors affecting childhood stunting is of paramount importance to investigate the effect of food and nutrition security strategies and suggest amendments [20]. Therefore, this study aimed to investigate the household food insecurity and identify sociodemographic factors affecting it among 2–6 years old children in an urban area in the southeast of Iran.

## 2. Materials and Methods

### 2.1. Study Design

A community-based cross-sectional survey was conducted from September to January 2018. This research was conducted in an urban area in the southeast of Iran with the highest rate of children malnutrition among Iranian households [9].

### 2.2. Participants

Children of both genders aged 2–6 years and their mothers (or their primary caretakers) were recruited. Given the role of mothers as their children’s primary caregiver, especially for feeding them, they were included in this research. On the other hand, unwillingness to take part in the study and the presence of any metabolic and congenital or chronic diseases in children led to their exclusion.

### 2.3. Data Collection

#### 2.3.1. Sampling

A multistage cluster sampling method was used based on probability proportional to size [21], through which 68 clusters were selected. From 6 different urban areas including Iranshahr, Nikshahr, Sarbaz, Fanuj, Dalgan, and Ghasreghand, the clusters were chosen using the table of random numbers. Next, information about the total number of 2–6 years old children in the selected clusters as the respective households was collected from the urban/rural health centers. In case of having two or more 2–6 years old children in the household, the youngest child was selected to avoid recall bias during data collection. Finally, participants were selected using systematic random sampling technique after identifying the first household randomly and proceeding to the second participant based on the determined interval.

For the sample size calculation, given a confidence level of 95%, a statistical power of 0.80, an effect size of 0.4, and a maximum linear estimate of 0.04% [22,23], the sample size was estimated 450 people. Therefore, data from 450 children aged 2–6 years from 6 different urban areas were collected by three members of the research team who were trained in educational workshops regarding the practical considerations of data collection for 2 consecutive days before taking part in the field study. The performance of the data collectors was monitored and evaluated by the principal investigator (M.S.) to ensure the quality and reliability of the data collection process.

#### 2.3.2. Instruments

Data about the sociodemographic characteristics of the participates including child’s gender and age, parent’s education level, parent’s occupation, access to drinking water, and sanitation as access to water closet (WC), disease history, birth weight, birth height, arm circumstance and also food security among households were collected through face-to-face interviews with the children’s mothers/primary caregivers.

Food insecurity among the households in the last 12 months was assessed using the 18-item U.S. Department of Agriculture (USDA) Core Food Security Module questionnaire [24]. It consists of food insecurity constructs of ‘quantity of food’, ‘quality of food’, ‘food acceptability’, and ‘certainty of getting food [24]. It has been translated to Farsi and validated in the Iranian context [25]. The questionnaire was filled out and accordingly, the food security status of each household was estimated based on the participants’ responses. Each response was coded as ‘affirmative’ or ‘negative’ using a two-choice scale of ‘yes’ or ‘no’. For those questions with more than two responses, ‘often’ and ‘sometimes’ was affirmative (value = 1), and ‘never’ was considered negative (value = 0). The coded responses were used to calculate an ordinal score in which 0 corresponded the most food-security households and 18 corresponded the most food-insecurity households. Finally, households were categorized into four groups using cutoff points proposed by Bickel et al. [24] as follows: ‘food secure households’ (0–2 affirmative responses); ‘food insecure without hunger households, which is equal to low food security [26]’ (3–7 affirmative responses); ‘food insecure with moderate hunger households, which is equal to moderate food insecurity [26]’ (8–12 affirmative responses); and ‘food insecure with severe hunger households, which is equal to very low food security [26]’ (13–18 affirmative responses). Five questions specifically referred to the children’s experiences for constructing a measure of children’s food insecurity across households. Three or more affirmative responses in the five child-specific questions indicated a household with child food insecurity. A cutoff point was selected to represent a reduction in the quality and quantity of food among children and to identify more serious forms of food insecurity than household-based ones [24,27].

The height of the children standing without shoes was measured with a precision of 0.1 cm using a tape measure mounted on a wall. Their weight with minimum clothing and without any shoes was measured using a calibrated scale (Seca^®^, Hamburg, Germany) with a precision of 0.1 kg.

After collecting anthropometric data, the WHO Anthro software was used to assess the children’s growth indices. The Z-scores of weight-for-age, height-for-age, and weight-for-height were calculated to determine the severity and prevalence of children’s underweight, stunting, and weight loss. The weight for age (underweight) index was divided into five categories: severely underweight (z < −3); underweight (−2 < z < −1); normal (−1 < z < +1); overweight (z > +2); and obese (z > +3). The height for age (stunting) index was divided into four categories: severely stunted (z < −3); stunting (−3 ≤ z ≤ −2); normal (−2 ≤ z ≤ +3); and tall stature (z > 3). The weight for height (wasting) index was divided into six groups: severely wasting (z < −3); wasting (−3 < z < −2); normal (−2 < z < +1); overweight (+1 < z < +2); obese (+2 < z < +3); and severely obese (z > +3).

#### 2.3.3. Ethical Considerations

The study protocol was approved by the Ethical Committee of Iranshahr University of Medical Sciences and Health Services, in which the first authors (M.S.) worked, under the code of IR.IRSHUMS.REC.1394.7. Furthermore, the mothers and the primary caregivers of the children were insured about their anonymity and confidentiality of collected data. An informed consent form was signed by those adults who willingly agreed to participate in the study.

#### 2.3.4. Data Analysis

Descriptive data analysis consisted of frequencies and proportions. Bivariate analysis estimated the crude effects of each covariate on the food security status. The chi-squared test was used to examine the correlation between qualitative variables. If a variable showed a significant association in the bivariate model, it was considered a candidate variable for inclusion to the multivariable model. The adjusted strength of each variable was examined using the multivariate ordinal logistic analysis. The odds ratio was calculated using the ordinal logistic regression model. The underlying assumption of ordinal logistic regression was parallel lines or proportional odds. Accordingly, the coefficients of variables associated with the probability of food security vs. food insecurity with depletion, and food security vs. inadequate intake and depletion, were constant [23]. The anthropometric and the logistic regression results were presented as proportions and adjusted odds ratios (AORs), respectively using 95% confidence intervals (CIs). All data analysis was performed using the SPSS software v.18 (IBM SPSS Inc., Chicago, IL, USA). *p* < 0.05 was considered statistically significant.

## 3. Results

Before the data analysis, some questionnaires were excluded due to unreliable (n = 4) and missing data (n = 25). To improve power of statistical analysis given the reduction of the sample size and enhance the ability to detect statistical significance, the effect size was changed to 0.1 indicating the minimum sample size required for data analysis in this study (n = 421); however, the confidence level, statistical power, and the maximum linear estimate remained unchanged [22,23]. Therefore, the data analysis was performed on data collected from 421 participants.

### 3.1. Sociodemographic Characteristics

The mean age of the children was 3.37 ± 1.07 years. The majority of them (54.1%) were male and in the age group of three years (35.4%). Moreover, 61.52%, 35.86%, and 2.7% of the children had normal weight, were underweight, and were overweight, respectively (Table 1).

### 3.2. Food Security Status and Weight for Age

The distribution of the children’s food security status by weight for age in the groups was reported in Table 1. Accordingly, 18.3% had food security, 2.6% had low food security, 9.2% had moderate food insecurity, and 69.9% had very low food security. Statistically significant relationships were observed between maternal education, access to drinking water and birth weight, with weight for age (*p* < 0.01).

Regarding weight, 76.6% of the children in the food security group, 90.9% in the low food security group, 69.2% in the moderate food insecurity group, and 55.4% in the very low food security group had normal weight (*p* < 0.01). Moreover, it was found that 53.7% of the children with literate mothers, 63.6% with mothers who had an under diploma education level, and 71.8% with mothers who had a diploma or higher education level had normal weight for age. In addition, 63.1% of the children who had access to drinking water had normal weight. On the other hand, 64.2% of the children who had birth weight more than 2500 g had normal weight and 54% of those with birth weight less than 2500 g were underweight.

### 3.3. Food Security Status and Height for Age

Mother’s education level, sanitation as access to WC, and birth weight were significantly associated with height for age (*p* < 0.01). Height for age was normal in 76.6% of the children in the food security group. Moreover, 63.6% of the children in the low food security group, 69.2% in the moderate food insecurity group, and 55.1% in the very low food security group had normal height (*p* < 0.01) (Table 2).

Moreover, 53% of the children with literate mothers, 62.9% with an under diploma education level, and 69.4% with a diploma or higher education level had normal height for age. It was found that 64.3% of the children who had access to WC were in the normal weight range, while 54.9% of the children who did not have any access to WC were stunted. Although 64% of the children who had birth weight more than 2500 g were in the normal height range, 58.7% of those who had birth weight less than 2500 g were stunted (*p* < 0.01).

### 3.4. Food Security Status and Weight for Height

There was a statistically significant relationship between sanitation as access to WC, birth height, and arm circumstance with weight for height (*p* < 0.01). Among the children with food security, 74% had normal weight for height. All children in the low food security group had normal weight for height. Moreover, 69.2% of the children in the moderate food insecurity group and 70.4% of the insecure children in the very low food security group were classified as normal weight for height (Table 3).

Moreover, 71.1% of the children who had access to WC and 74% of the children without it had normal weight for height. It was reported that 71.7% of the children with birth height more than 45 cm and 72.4% with birth height less than 45 cm had a normal weight for height. Overall, 72.5% of the children with arm circumference greater than 11.5 cm had normal weight, but 50% of the children with arm circumference less than 11.5 cm were wasted (*p* < 0.01).

### 3.5. The Logistic Regression Model of Food Security

The model of ordinal logistic regression showed statistically significant relationships between the children’s food security and weight for age. The weight gain was 2.63 times lower in the children in the low food insecurity group than in those children with normal food security (95% confidence interval: −4.952, −1.41). There was no statistically significant relationship between the children in the moderate food insecurity group and the very low food security group with regard to weight for age (Table 4).

Converting the children’s food security status from normal food insecurity to low food security group and moderate food insecurity showed that the chance of height rise was less, i.e., 1.91 times (95% confidence interval: −3.66, −1.01) and 1.41 times (95% confidence interval: −3.45, 0.69), respectively. Moreover, no statistically significant relationship was reported between the children in the very low food security group and height for age (Table 5).

In addition, there was no statistically significant relationship between the children’s food security status and weight for height in any of the food security groups (Table 6).

## 4. Discussion

This research was conducted to investigate the status of household food insecurity and sociodemographic factors affecting it among 2–6 years old children in an urban area in the southeast of Iran.

In this study, statistically significant relationships were observed between household food insecurity and underweight, stunting, and wasting. A study in Colombia showed that children with food insecurity households were three times more likely to lose weight, but stunting had no association with food insecurity in the family [28]. In contrast, a study in South Korea showed a significant relationship between food insecurity and being overweight [11]. Weight gain among infants and toddlers continues to be a significant concern, especially in low-income households [29]. However, these controversial findings appear to be partly due to differences in the module used to assess the families’ food security status, age group of samples, reference used to determine the children’s nutritional status, and the studies’ sample size. According to the WHO’s malnutrition classification, stunting, underweight, and weight loss are considered either very serious or critical, or high or serious in communities with the percentages of 30–39.9%, 20–29.9%, and 10–14.9%, respectively [30]. In our study, the prevalence of malnutrition in terms of underweight and stunting was in the very high range and weight loss was in the critical range. The prevalence of mild, moderate, and severe household food insecurity was 2.6%, 9.2%, and 69.9%, respectively. The prevalence of moderate and severe household food insecurity in Nepal, Haromaya District of Ethiopia, Kailali District of Nepal, and Tabriz city in Iran has been reported 19.0–23.2%, 39.7%, 69%, and 30%, respectively [31,32,33,34], which are less than the reported rate in our study. However, the interpretation of these differences should be done with caution because the same module for the measurement of food insecurity has not been used in these studies. Moreover, other studies in different provinces of Iran have reported various degrees of food insecurity depending on growth and development conditions. For instance, a study in Isfahan city showed that the prevalence of food insecurity among households aged 14–17 years was 36.6% [35]. The results of the food security study in Yazd city indicated that 32.9% of households aged 6–12 years had food insecurity [36].

In our study, food insecurity had the same condition among males and females, which was consistent with the finding of a study in Burkina Faso [37]. Moreover, food insecurity was prevalent and was associated with socio-demographic factors among households with pre-school children in southeastern Iran and Ethiopia [17,38]. In contrast, studies in other regions showed that the mean score of food security could be higher among females [36,37]. In developing countries, differences have been found in the allocation of household resources by location within the household. Therefore, women are mostly found to be disadvantaged due to the culture of less valuing them than men [38]. Gender bias may also have been reflected in the allocation of food resources for children [39].

In the present study, a negative association was found between the mother’s education level and the food security status as households with mothers having a lower education level had worse food security. However, no such an association was observed between the father’s education level and food security status. Conversely, in the international literature, a significant relationship has been found between the father’s job status and food insecurity [17,40]. A study in Malaysia reported that households with working mothers usually had greater food expenditure and a higher degree of food security, because of their contribution to total household income [41]. The influence of food security on the nutritional status of children is also mediated by the mother’s knowledge of child nutrition, education, healthy environmental conditions, and sanitation. For instance, a research in Bushehr in the south of Iran showed that more than half of children whose mothers had a diploma or higher education level had normal weight for age and promoting the mothers’ level of education might enhance food security in the family [42]. A study in Pakistan found that the prevalence of stunting among children under five years was nearly 40% in households where all of adult women were illiterate. This figure decreased to 25% when at least one adult woman had completed at least 10 years of schooling [43].

In terms of sanitation, about 55% of the children in our study with no access to WC were stunted. Supporting this finding, a study in Uganda showed that water and sanitation practices were associated with household food insecurity. Accordingly, not owning a toilet increased the likelihood of being food insecure [44].

Our findings showed a significant rate of very low food security among the households, which was against the findings of similar studies in other areas of Iran including Isfahan 7.3% [35], Yazd 3.7% [36], and Bushehr 11.3% [42], as well as in neighbor countries such as Pakistan 24.3% [43]. The discrepancy of prevalence rate may be due to the geographical conditions of the regions as the research zone of our study has a subtropical hot desert climate, sample size, age of samples included in the studies, and economical and cultural differences between the research areas.

Since data was collected using self-report questionnaires via interviews, non-response, and recall bias could be a limitation of our study. Moreover, cause-and-effect relationships could not be detected using data collected from this cross-sectional survey and our findings do not reflect the food security condition in other provinces of Iran. In addition, in studies about health and quality of life, missing and inconsistent data are common in data collection especially from people with a low educational level or those living in socio-economically deprived areas [45]. Similarly, some of the questionnaires were excluded from the data analysis in our study and they could not be replaced through taking samples from new households because of funding limitations. However, our study can provide valuable and reliable data on food insecurity among households with children aged 2–6 years in the southeast of Iran. Future studies are needed to determine how resources can be allocated or what political steps are necessary to overcome this nutrition crisis at the national level, which can have practical implications for other developing and transitional countries with the similar nutritional status.

## 5. Conclusions

This study showed that food insecurity in various levels was prevalent in households with children aged 2–6 years living in southeast of Iran. Specifically, weight and height, mother’s education level, and sanitation as access to WC were associated with food insecurity.

Since early childhood is the most important developmental phase in the human’s life, designing and implementing strategies to improve access to food and sanitation, and enhance maternal nutritional knowledge should be considered by policy-makers to enhance food security and quality of life among children aged 2–6 years. 

## Figures and Tables

**Table 1:** The participants’ sociodemographic characteristics by weight for age.

Variable	N (%)	Weight for Age			*p* Value
		Underweight N (%)	Normal N (%)	Overweight N (%)	
Gender					
Female	193 (45.9)	70 (36.3)	121 (62.7)	2 (1.0)	0.17
Male	228 (54.1)	81 (35.5)	138 (60.5)	9 (4.0)	
Father’s education level					
Illiterate	120 (285)	51 (42.5)	67 (55.8)	2 (1.7)	0.23
Under diploma	200 (45.5)	72 (36.0)	122 (61.0)	6 (3.0)	
Diploma/academic	101 (24.0)	28 (27.7)	70 (69.3)	3 (3.0)	
Mother‘s education level					
Illiterate	149 (35.4)	66 (44.3)	80 (53.7)	3 (2.0)	<0.01
Under diploma	187 (44.4)	66 (35.3)	118 (63.1)	3 (1.6)	
Diploma/academic	85 (20.2)	19 (22.4)	61 (71.8)	5 (5.9)	
Access to drinking water					
Yes	363 (86.2)	128 (35.3)	229 (63.1)	6 (1.7)	<0.01
No	58(13.8)	23 (39.7)	30 (51.7)	5 (8.6)	
Access to WC					
Yes	339 (80.5)	118 (34.8)	214 (63.1)	7 (2.1)	0.19
No	82 (19.5)	33 (40.2)	45 (54.9)	4 (4.9)	
Background disease					
Yes	61 (14.5)	28 (45.9)	33 (54.1)	0 (0.0)	0.10
No	360 (85.5)	123 (34.2)	226 (62.8)	11 (3.1)	
Birth weight					
>2500	358 (85.0)	117 (32.7)	230 (64.2)	11 (3.1)	<0.01
<2500	63 (15.0)	34 (54.0)	29 (46.0)	0 (0.0)	
Household food security status					
Normal food security	77 (18.3)	14 (18.2)	59 (76.6)	4 (5.2)	<0.01
Low food security	11 (2.6)	1 (9.1)	10 (90.9)	0 (0.0)	
Moderate food insecurity	39 (9.2)	9 (23.1)	27 (69.2)	3 (7.7)	
Very low food security	294 (69.9)	127 (43.2)	163 (55.4)	4 (1.4)	

WC: water closet.

**Table 2:** The participants’ sociodemographic characteristics by height for age.

Variable	N (%)	Height for Age			*p* Value
		Stunting N (%)	Normal N (%)	Tall N (%)	
Gender					
Female	193 (45.9)	69 (35.8)	123 (63.7)	1 (5.0)	0.28
Male	228 (54.1)	92 (40.4)	132 (57.9)	4 (1.8)	
Father’s education level					
Illiterate	120 (285)	55 (45.8)	64 (53.3)	1 (0.8)	0.06
Under diploma	200 (45.5)	76 (38.0)	120 (60.0)	4 (2.0)	
Diploma/academic	101 (24.0)	30 (29.7)	71 (70.3)	0 (0.0)	
Mother‘s education level					
Illiterate	149 (35.4)	69 (46.3)	79 (53.0)	1 (0.7)	0.01
Under diploma	187 (44.4)	69 (36.9)	117 (62.9)	1 (0.5)	
Diploma/academic	85 (20.2)	23 (27.1)	59 (69.4)	3 (3.5)	
Access to drinking water					
Yes	363 (86.2)	134 (36.9)	224 (61.7)	5 (1.4)	0.27
No	58 (13.8)	27 (46.6)	31 (53.4)	0 (0.0)	
Access to WC					
Yes	339 (80.5)	116 (34.2)	218 (64.3)	5 (1.5)	<0.01
No	82 (19.5)	45 (54.9)	37 (45.1)	0 (0.0)	
Background disease					
No	360 (85.5)	135 (37.5)	220 (61.1)	5 (1.4)	0.51
Yes	61 (14.5)	26 (42.6)	35 (57.4)	0 (0.0)	
Birth weight					
≥2500 g	358 (85.0)	124 (34.6)	229 (64.0)	5 (1.4)	<0.01
<2500 g	63 (15.0)	37 (58.7)	26 (41.3)	0 (0.0)	
Birth height					
≥45 cm	392 (93.1)	145 (37.0)	242 (61.7)	5 (1.3)	0.13
<45 cm	29 (6.89)	16 (55.2)	13 (44.8)	0 (0.0)	
Household food security status					
Normal food security	77 (18.3)	16 (20.8)	59 (76.6)	2 (2.6)	0.01
Low food security	11 (2.6)	4 (36.4)	7 (63.6)	0 (0.0)	
Moderate food insecurity	39 (9.2)	12 (30.8)	27 (69.2)	0 (0.0)	
Very low food security	294 (69.9)	129 (43.9)	162 (55.1)	3 (1.0)	

**Table 3:** The participants’ sociodemographic characteristics by weight for height.

Variable	N (%)	Weight for Height				*p* Value
		Wasting N (%)	Normal N (%)	Overweight N (%)	Obese N (%)	
Gender						
Female	193 (45.9)	40 (20.7)	143 (74.1)	8 (4.1)	2 (1.0)	0.77
Male	228 (54.1)	57 (25.0)	159 (69.7)	10 (4.4)	2 (0.9)	
Father’s education						
Illiterate	120 (285)	21 (17.5)	92 (76.7)	6 (5.0)	1 (0.8)	0.71
Under diploma	200 (45.5)	53 (.26.5)	138 (69.0)	7 (3.5)	2 (1.0)	
Diploma/academic	101 (24.0)	23 (22.8)	72 (71.3)	5 (5.0)	1 (1.0)	
Maternal education						
Illiterate	149 (35.4)	34 (22.8)	107 (71.8)	7 (4.7)	1 (0.7)	0.68
Under diploma	187 (44.4)	46 (24.6)	131 (70.1)	9 (4.8)	1 (0.5)	
Diploma/university	85 (20.2)	17 (20.0)	64 (75.3)	2 (2.4)	2 (2.4)	
Access to drinking water						
Yes	363 (86.2)	83 (22.9)	264 (72.7)	13 (3.6)	3 (0.8)	0.28
No	58 (13.8)	14 (24.1)	38(65.5)	5 (8.6)	1 (1.7)	
Access to WC						
Yes	339 (80.5)	85 (25.1)	241 (71.1)	12 (3.5)	1 (0.3)	<0.01
No	82 (19.5)	12 (14.6)	61 (74.4)	6 (7.3)	3 (3.7)	
Background disease						
No	360 (85.5)	79 (21.9)	260 (72.2)	17 (4.7)	4 (1.1)	0.36
Yes	61 (14.5)	18 (29.5)	42 (68.9)	1 (1.6)	0(0.0)	
Birth weight						
≥2500 g	358 (85.0)	84 (23.5)	254 (70.9)	17 (4.7)	3 (0.8)	0.58
<2500g	63 (15.0)	13 (20.6)	48 (76.2)	1 (1.6)	1 (1.6)	
Birth height						
≥45 cm	392 (93.11)	93 (23.7)	281 (71.7)	16 (4.1)	2 (0.5)	<0.01
<45 cm	29 (6.89)	4 (13.8)	21 (72.4)	2 (6.9)	2 (6.9)	
Arm circumference						
≥11.5 cm	415 (98.6)	94 (22.7)	301 (72.5)	18 (4.3)	2 (0.5)	<0.01
<11.5	6 (1.4)	3 (50.0)	1 (16.7)	0 (0.0)	2 (33.3)	
Household food security status						
Normal food security	77 (18.3)	17 (22.1)	57 (74.0)	2 (2.6)	1 (1.3)	0.04
Low food security	11 (2.6)	0 (0.0)	11 (100.0)	0 (0.0)	0 (0.0)	
Moderate food insecurity	39 (9.2)	8 (20.5)	27 (69.2)	3 (7.7)	1 (2.6)	
Very low food security	294 (69.9)	72 (24.5)	207 (70.4)	13 (4.4)	2 (0.7)	

**Table 4:** Odds ratios for weight for age based on the ordinal logistic regression model.

Variable	N	Adjusted Odds Ratio *	95% Confidence Interval	*p* Value
Household food security status				
Normal food security	77	1	-	
Low food security	11	−2.63	−4.95, −1.41	<0.01
Moderate food insecurity	39	−1.10	−2.74, 2.24	0.58
Very low food security	294	1.47	−2.97, 6.48	0.60
Mother’s education level				
Illiterate	149	1	-	
Under diploma	187	−2.09	−3.93, −1.09	0.02
Diploma/academic	85	−1.76	−3.12, −1.06	0.08
Birth weight				
≥2500 g	358	1	-	
<2500 g	63	−1.20	−3.89, −1.24	<0.01
Access to drinking water				
Yes	363	1	-	
No	58	−1.15	−2.09, 1.55	0.62

* The odds ratios were adjusted for mother’s education level, birth height, and access to WC.

**Table 5:** Odds ratios of height for age based on the ordinal logistic regression model.

Variables	N	Adjusted Odds Ratio *	95% Confidence Interval	*p* Value
Household food security status				
Normal food security	77	1	-	
Low food security	11	−1.91	−3.66, −1.01	0.04
Moderate food insecurity	39	−1.41	−3.45, 0.69	0.02
Very low food security	294	−1.46	−5.52, 2.58	0.57
Father’s education level				
Illiterate	120	1	-	
Under diploma	200	1.00	−1.89, 1.82	0.98
Diploma/academic	101	1.12	−1.52, 1.89	0.65
Mother’s education level				
Illiterate	149	1	-	
Under diploma	187	1.93	−3.78, 1.00	0.05
Diploma/academic	85	1.55	−2.85, 1.18	0.15
Birth weight				
≥2500 g	358	1	-	
<2500 g	63	−2.31	−4.17, −1.29	<0.01
Arm circumference				
≥11.5 cm	415			
<11.5	6	−2.41	−4.65, 0.45	0.44
Access to WC				
Yes	339	1	-	
No	82	−1.97	−3.32, −1.17	<0.01

* The odds ratios were adjusted for father’s education level, mother’s education level, birth height, arm circumference, and access to WC.

**Table 6:** Odds ratio of weight for height based on the ordinal logistic regression model.

Variables	N	Adjusted Odds Ratio *	95% Confidence Interval	*p* Value
Household food security status				
Normal food security	77	1	-	
Low food security	11	1.25	−1.39, 2.20	0.42
Moderate food insecurity	39	2.27	−1.82, 9.48	0.25
Very low food security	294	1.64	−1.30, 3.56	0.20
Birth height				
≥2500 g	392	1	-	
<2500 g	29	1.05	−1.73, −1.91	0.87
Arm circumference				
≥11.5 cm	415	1	-	
<11.5	6	1.08	−2.43, 5.58	0.92
Access to WC				
Yes	339	1	-	
No	82	2.38	1.30, 4.30	<0.01

* The odds ratios were adjusted for birth height, arm circumference, and access to WC.

## Data Availability

The data presented in this article is available on reasonable request from the corresponding author.
